# Bright light increases blood pressure and rate‐pressure product after a single session of aerobic exercise in men

**DOI:** 10.14814/phy2.16141

**Published:** 2024-07-18

**Authors:** Gustavo F. Oliveira, Thais C. Marin, Julio C. C. L. Barbosa, Luan M. Azevêdo, Saurabh S. Thosar, José Cipolla‐Neto, Claudia L. M. Forjaz, Leandro C. Brito

**Affiliations:** ^1^ Chronobiology and Exercise Physiology Applied Research Group School of Arts and Science, University of São Paulo São Paulo Brazil; ^2^ Exercise Hemodynamic Laboratory School of Physical Education and Sport, University of São Paulo São Paulo Brazil; ^3^ Oregon Institute of Occupational Health Sciences, Oregon Health & Science University Portland Oregon USA; ^4^ Neurobiology Laboratory Institute of Biomedical Science, University of São Paulo São Paulo Brazil

**Keywords:** exercise, heart rate, hemodynamic, light, postexercise hypotension

## Abstract

This study aimed to test whether bright light (BL) exposure attenuates the reduction in blood pressure (BP) postexercise compared to dim light (DL). Twenty healthy men (27 ± 5 years) randomly underwent two experimental sessions: one under BL (5000 lux) and another under dim light (DL <8lux). In each session, subjects executed a bout of aerobic exercise (cycle ergometer, 30 min, moderate intensity). BP (oscillometric) and heart rate (HR monitor) were measured, and rate‐pressure‐product (RPP) was calculated. Additionally, a 24‐h ambulatory blood pressure monitoring (ABPM) was conducted after the sessions. Systolic BP decreased while HR increased significantly and similarly after the exercise in both sessions. Additionally, systolic BP levels were higher in BL than DL throughout the experimental session (*P*_session_ = 0.04). Diastolic (*P*_interaction_ = 0.02) and mean (*P*_interaction_ = 0.03) BPs decreased after exercise in DL (at 30 min), and increased in BL (at 60 and 90 min). RPP increased in both sessions postexercise, but with a main effect revealing higher levels throughout the experimental session in BL than DL (*P*_session_ = 0.04) and during the first 3 h of ABPM (*p* = 0.05). In healthy men, BL exposure increased systolic BP and cardiac work, and abolished the postexercise decreases of diastolic and mean BPs.

## Graphical abstract

Bright light exposure abolished the postexercise hypotension observed for diastolic and mean blood pressures (BPs) compared to dim light exposure. In addition, bright light promoted greater values of systolic BP and cardiac workload throughout the experimental session. Arrows indicate directions of a variable change after aerobic exercise compared to pre‐exercise values under the two light conditions: ↑ (increase); ↓ (decrease). Created with Biorender.

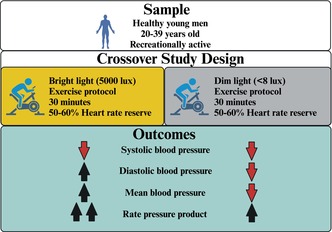

## 1 INTRODUCTION

A single session of aerobic exercise decreases blood pressure (BP) during the postexercise period in comparison to the values obtained before the exercise, a phenomenon known as postexercise hypotension (PEH) (Kenney & Seals, 1993). PEH holds clinical relevance due to its significant magnitude and long‐lasting duration (Brito, Fecchio, et al., 2018; Kenney & Seals, 1993). Additionally, PEH magnitude correlates with the chronic BP‐lowering effect of exercise training in patients not receiving anti‐hypertensive medication (Hecksteden et al., 2013; Liu et al., 2012), which may increase its practical applicability. However, PEH magnitude varies across studies from −9 to −5 and from −11 to −1 mmHg for systolic and diastolic BPs, respectively (Carpio‐Rivera et al., 2016). Similarly, the duration of PEH varies between 20 min and 16 h across studies (Brito et al., 2019). Differences regarding the experimental protocols may explain, at least in part, this expressive variability (Brito et al., 2019).

Previous studies have shown that light intensity can induce cardiovascular adjustments. Along this line, exposure to bright light (BL) may affect BP when subjects remain at rest. For instance, exposure to BL (i.e., 2800 lux) for 4 h increased systolic BP compared to dim light (DL) of 120 lux during the biological night (Yokoi et al., 2006). In another study, 25 min of exposure to 5000 lux increased muscle sympathetic nerve activity, which suggests an increase in vascular resistance, although changes in BP were not observed (Saito et al., 1996). Similarly, cardiac sympathetic modulation and heart rate (HR) also increased after 1 h of exposure to 5000 lux (Sakakibara et al., 2000; Scheer et al., 2004). These findings suggest that BL increases sympathetic activity and, consequently, HR and BP.

A reduction of muscular sympathetic activity after exercise, leading to the maintenance of exercise‐induced vasodilation during the recovery period, is accepted as a possible mechanism of PEH occurrence (Halliwill et al., 1996). Thus, since BL may increase vascular resistance via sympathetic activity, it is possible to hypothesize that exposure to BL could attenuate PEH in magnitude and duration. To test this hypothesis, the current study was designed to investigate whether BL (5000 lux) increases BP at rest and affects PEH assessed inside the laboratory and under ambulatory conditions in comparison with DL light (<8 lux).

## 2 METHODS

Data were collected between May 2021 and June 2022. Healthy men were included if they were aged between 20 and 39 years old, were nonsmokers, were not taking any over‐the‐counter medication or vitamin supplements, and reported no previous diagnosis of chronic diseases. Accomplishment with these requirements was verified by self‐reporting and the fulfillment of the Physical Activity Readiness Questionnaire (Shephard et al., 1981). Additionally, subjects underwent the following preliminary exams to confirm their eligibility for the study's criteria. Resting BP was measured in two different visits, and in each visit, measures were taken three times on both arms after 5 min of seated rest. Subjects with average values >140 and/or >90 mmHg for systolic and diastolic BPs, respectively, were excluded (Pescatello et al., 2004). Body weight and height were measured, body mass index (BMI) was calculated, and the subjects with BMI >30 kg/m^2^ were excluded (National Institute of Health, 2000). Chronotype status was assessed using Horne and Ostberg's morningness–eveningness questionnaire (Horne & Ostberg, 1976). Subjects with morningness (>65 scores) or eveningness chronotypes (<45 scores) were excluded. Physical activity levels were assessed using the short version of the International Physical Activity Questionnaire (Craig et al., 2003), and subject >300 min/week of moderate‐intensity or >60 min/week of vigorous‐intensity were excluded.

All subjects signed an informed written consent before enrollment. The study was approved by the Research Ethical Committee of the School of Physical Education and Sport of the University of São Paulo (3.742.479). It was registered at the Brazilian Clinical Trials (http://ensaiosclinicos.gov.br/rg/RBR‐4vcfjmm).

### 2.1 Study design

The study followed a randomized crossover design. All subjects underwent two experimental sessions (BL and DL) in a random order (simple randomization). The experimental sessions were conducted on the same day of the week with an interval of 7 days between them. Preceding each experimental session, subjects were instructed to keep similar routines, including bedtime and wake time. They were also asked to abstain from physical efforts and caffeinated or alcoholic beverages for 24 h. In addition, they should be fastened for at least 2 h before reporting to the laboratory. Adherence to these instructions was checked by self‐report.

**FIGURE 1:**
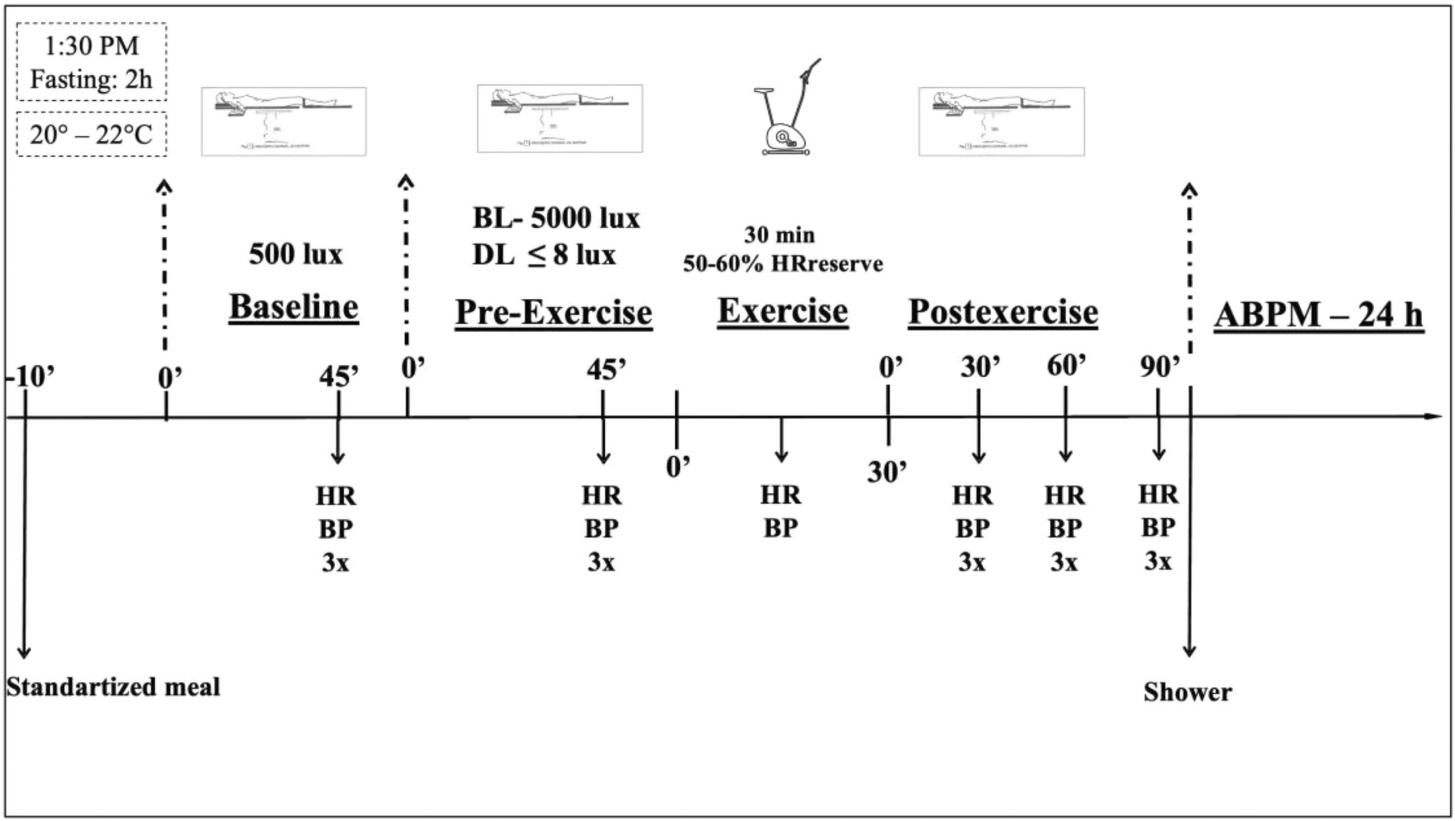
Experimental Session. ABPM, ambulatory blood pressure monitoring; BL, bright light; BP, blood pressure; DL, dim light; HR, heart rate.

The experimental sessions were conducted in a temperature‐controlled laboratory (20 to 22°C), composed of four periods (baseline, pre‐exercise, exercise, and postexercise). Afterward, subjects were instructed to wear an ambulatory blood pressure monitoring (ABPM). Subjects reported to the laboratory at 1:30 pm, received a standardized meal (2 cereal bars and 100 mL of water), and had a bathroom opportunity as done in previous studies (Brito et al., 2015; Brito, Rezende, et al., 2018). Then, they lay down on a bed under a wash‐out light condition of 500 lux. BP and HR were measured in triplicate at 45 min, and the average of these measures was considered the baseline assessment. Then, the light intensity was adjusted to BL (5000 lux) or DL (<8 lux) in accordance with the experimental session and remained at this intensity until the end of the session. Initially, the subjects remained supine under this new light condition. BP and HR were measured again in triplicate at 45 min, and the average of these measures was considered a pre‐exercise assessment. Afterward, the subjects moved to the cycle ergometer and exercised for 40 min, returning to the supine position immediately after the exercise. Then, the triplicate measurements of BP and HR were repeated at 30, 60, and 90 min after the exercise, and the mean value of each moment was calculated for postexercise assessments. Finally, the subjects showered, and the 24‐h ABPM started at 6:00 pm (Figure 1).

### 2.2 Aerobic exercise

The exercise bout executed in both experimental sessions was preceded by a 5‐min warm‐up and followed by a 5‐min cool‐down period, both pedaling with 30 watts. During the exercise, the subjects pedaled for 30 min at 50%–60% of their HR reserve calculated according to Karvonen's formula (Karvonen et al., 1957) and using the maximal HR predicted for their age. HR was continuously measured during the exercise (Polar, CRX800, Kempele, Finland), and workload was adjusted to maintain the desired HR. BP was measured at 15 min of exercise by the auscultatory method using an aneroid sphygmomanometer (Missouri, Mikato Ltda, São Paulo, Brazil).

### 2.3 Light settings

**FIGURE 2:**
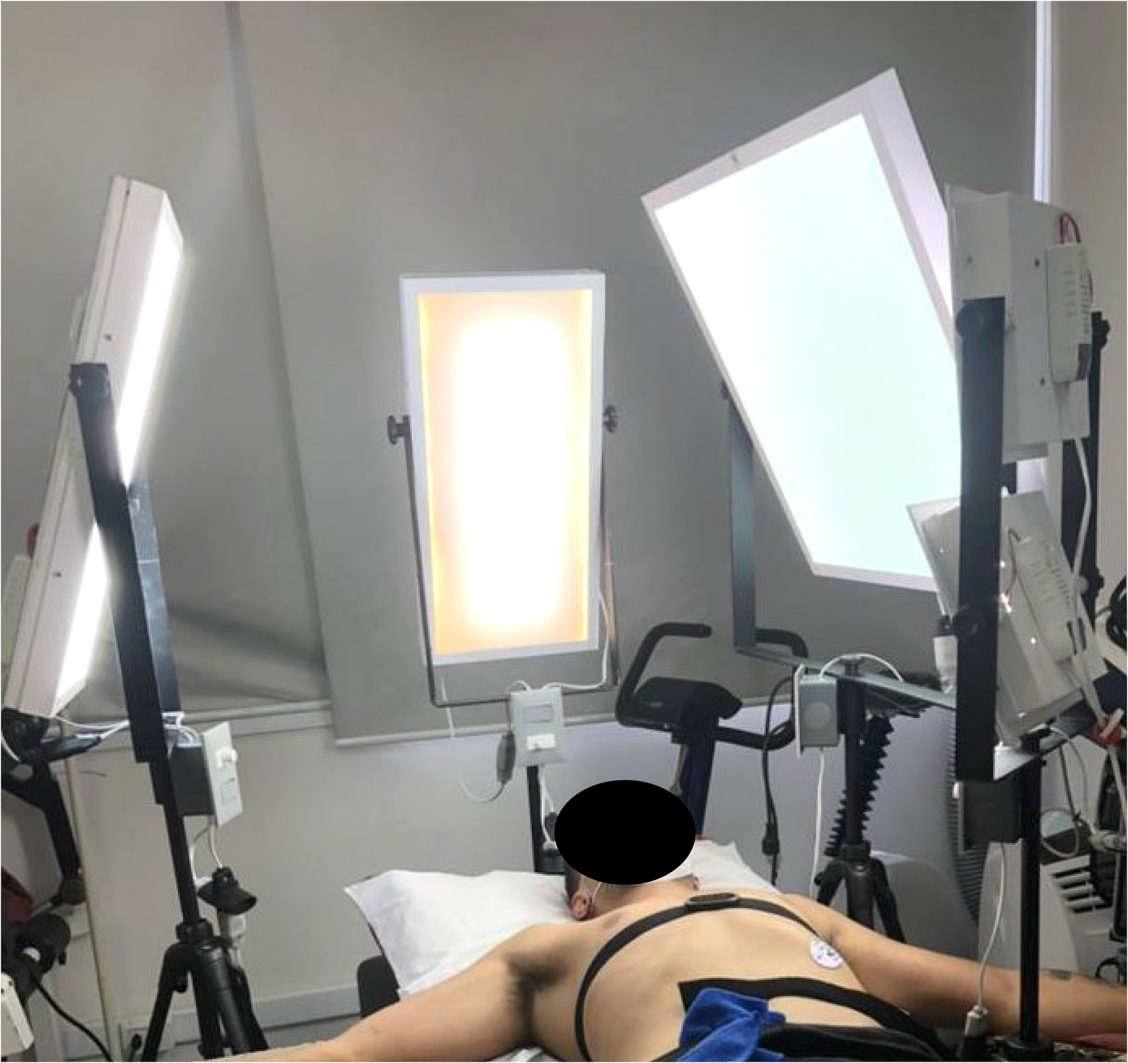
Demonstrative figure of light set up in the laboratory.

Light intensity was controlled using customized polychromatic LED lamps positioned around the subjects and directed to their eyes (Figure 2). LED lamps adjusted for BL condition exhibited: 5000 lux; correlated color temperature (CCT) = 5022 K; and average irradiance level and wavelength for blue = 10.4 μW/cm^2^ and 450 nm, green = 6.7 μW/cm^2^ and 550 nm, yellow = 7.8 μW/cm^2^ and 580 nm, orange = 7.1 μW/cm^2^ and 600 nm, and red = 2.3 μW/cm^2^ and 700 nm. DL (≤8 lux) was conducted with the lamps turned off. During the experiments, the light intensity was measured by a lux meter (Insthrumental 920 T, São Paulo, Brazil) whenever the light intensity was changed. To increase the accuracy, celling laboratory lamps were turned off, and blackout curtains blocked any external light to allow only the customized lamps to control the light intensity.

### 2.4 Measurements

During the experimental sessions, BP was measured on the subjects' right arm using an oscillometric device (Omron, Elite+ HEM‐7320, Kioto, Japan) and HR by an HR monitor (Polar, CRX800, Kempele, Finland). Rate‐pressure‐product (RPP) was calculated by the product of systolic BP and HR.

For ABPM, an oscillometric device (Spacelabs 90,207; Spacelabs, Inc., Redmond, WA) was positioned on subject's nondominant arm. Measurements were taken every 15 min for 24 h. During this period, the subjects were instructed to keep their usual daily activities and avoid exercise, alcoholic and caffeinated beverages. Adherence to these instructions was confirmed by verbal self‐reporting and daily diary completion. Only recordings with more than 80% of successful measures were analyzed. The averages of HR, systolic, diastolic, and mean BPs, and RPP were calculated for the following periods: 24‐h (i.e., all measures), awake (i.e., all measures obtained during the period the subjects reported to be awake), and asleep (i.e., all measures obtained during the period the subjects reported to be sleeping). Additionally, the values measured during the first 3 h of monitoring (a period in which all subjects were still awake) were averaged.

### 2.5 Data analysis

For this study design, a minimal sample size of 18 subjects was determined (G*Power v. 3.1.9.2, Universität Kiel, Germany) as necessary to get a power of 80% when considering an α error of 0.05 and an effect size Cohens f of 0.30 for systolic BP (Brito et al., 2015).

Normal data distribution was checked using the Shapiro–Wilk test, and homogeneity was assessed using Levene's test.

To compare the effects of light intensities at rest, two‐way ANOVAs for repeated measures were used, employing session (BL vs. DL) and time (baseline vs. pre‐exercise) as the main factors. To compare the effects of light intensity on responses to exercise, two‐way ANOVAs for repeated measures were also employed, considering session (BL vs. DL) and time (pre‐exercise vs. 30, 60, and 90 min postexercise) as main factors. Post‐hoc analyses were conducted using Newman–Keuls test when necessary.

Paired *t*‐tests were used to compare the effects of light intensity on ABPM averages.

The statistical analyses were conducted using a specific software package (Statsoft v.14 Statistic for Windows, Tulsa, USA). *p* ≤ 0.05 was set as significant for all analyses. Data are shown as mean ± standard deviation.

## 3 RESULTS

Twenty‐three subjects signed the written consent. One subject was excluded due to BP values above the study criteria, and another due to BMI >30 kg/m^2^. Thus, 21 subjects initiated the experimental protocol, but one dropped out due to personal reasons. The characteristics of the final sample are shown in Table 1.

**TABLE 1:** Subjects' characteristics.

	Values
*N*	20
Age (years)	28 ± 4
Chronotype (scores)	48 ± 10
Anthropometric	
Height (m)	1.77 ± 0.05
Weight (kg)	77.6 ± 13.0
Body mass index (kg/m^2^)	24.7 ± 3.5
Hemodynamic	
Systolic blood pressure (mmHg)	115 ± 8
Diastolic blood pressure (mmHg)	76 ± 6
Mean blood pressure (mmHg)	89 ± 6
Heart rate (bpm)	72 ± 13
Physical activity level	
Inactive, *n* (%)	3 (15)
Insufficiently active, *n* (%)	8 (40)
Active, *n* (%)	9 (45)

*Note*: Values in mean ± SD.

Comparisons between baseline and pre‐exercise periods are shown in Table 2. Systolic BP did not change from the baseline to the pre‐exercise period in any of the sessions (*P*_interaction_ = 0.22). Diastolic and mean BP increased similarly in BL and DL sessions (+4 ± 3 vs. +5 ± 5 mmHg, *P*_time_ <0.01 and + 3 ± 3 vs. +4 ± 4 mmHg, *P*_time_ <0.01), respectively. HR and RPP decreased similarly in BL and DL sessions (−2 ± 3 vs. −2 + 3 bpm, *P*_time_ <0.01 and −172 ± 453 vs. −115 ± 497 mmHg × bpm, *P*_time_ <0.01), respectively.

**TABLE 2:** Hemodynamic variables assessed at rest in both experimental sessions, bright light (BL) and dim light (DL).

	Baseline	Pre‐exercise	*Ps*	*Pt*	*P s x t*
Systolic BP (mmHg)					
BL	122 ± 11	122 ± 11	0.53	0.20	0.60
DL	120 ± 12	122 ± 8			
Diastolic BP (mmHg)					
BL	67 ± 7	71 ± 6^a^	0.40	<0.01	0.30
DL	66 ± 7	71 ± 7^a^			
Mean BP (mmHg)					
BL	85 ± 7	88 ± 6^a^	0.09	0.03	0.64
DL	83 ± 7	87 ± 5^a^			
Heart rate (bpm)					
BL	63 ± 9	61 ± 11^a^	0.25	< 0.01	0.58
DL	62 ± 8	60 ± 8^a^			
Rate‐pressure‐product (mmHg × bpm)					
BL	7740 ± 1544	7568 ± 1580^a^	0.58	0.01	0.75
DL	7491 ± 1171	7376 ± 1132^a^			

*Note*: Data are shown as mean ± SD. Abbreviations: BP, blood pressure; *s*, session; *t*, time.

^a^ Significantly different from baseline (*p* ≤ 0.05).

The exercise was executed with similar workloads in the BL and PN sessions (102 ± 23 vs. 101 ± 25 watts, respectively, *p* = 0.74) and similar intensities (BL = 53 ± 7% and DL = 54 ± 6% of the HR reserve, *p* = 0.83). Hemodynamic responses between pre‐exercise and exercise periods in both experimental sessions are shown in Table 3. During the exercise, systolic BP and HR increased similarly in both sessions in comparison to the pre‐exercise values (both *P*_time_ <0.01), while diastolic BP remained unaltered (*P*_interaction_ = 0.69).

**TABLE 3:** Hemodynamic responses assessed Pre‐exercise and during Exercise in the bright light (BL) and dim light (DL) sessions.

	Pre‐exercise	Exercise	*Ps*	*Pt*	*P sxt*
Systolic BP (mmHg)					
BL	122 ± 11	151 ± 14^a^	0.89	<0.01	0.10
DL	122 ± 11	152 ± 12^a^			
Diastolic BP (mmHg)					
BL	71 ± 6	72 ± 7	0.49	0.23	0.69
DL	71 ± 7	71 ± 6			
Heart rate (bpm)					
BL	61 ± 11	126 ± 8^a^	0.25	<0.01	0.06
DL	60 ± 8	127 ± 7^a^			

*Note*: Data are shown as mean ± SD. Abbreviations: BP, blood pressure; *s*, session; *t*, time.

^a^ Significantly different from pre‐exercise (*p* < 0.05).

**FIGURE 3:**
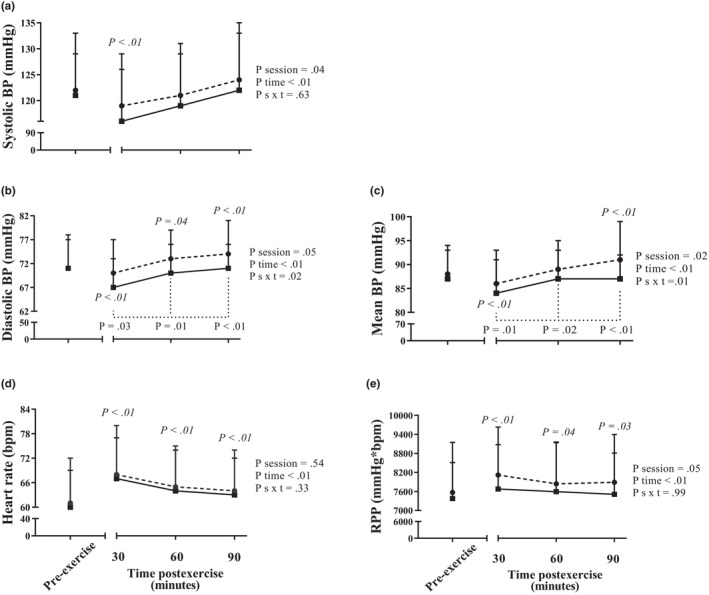
Systolic, diastolic, and mean blood pressures, heart rate, and rate‐pressure‐product assessed pre‐exercise and at 30, 60, and 90 min postexercise during bright light session (represented by dashed lines with circles) and dim light session (represented by continuous lines with squares). *p* values in *Italics* indicate a pairwise difference from pre‐exercise in the same session. *p* values under dashed lines indicate pairwise difference from BL at the same time postexercise. Data are shown as mean ± SD. BP, blood pressure; RPP, rate‐pressure‐product.

Hemodynamic responses between pre‐ and postexercise in both experimental sessions are shown in Figure 3. In comparison with pre‐exercise, systolic BP decreased similarly at 30 min of the postexercise period and returned to the pre‐exercise level at 60 and 90 min in both BL and DL sessions (*P*_time_ <0.01). Regardless of the moment, systolic BP levels were higher in BL than DL (*P*_session_ = 0.04). Diastolic and mean BPs decreased significantly in comparison to pre‐exercise value at 30 min of the postexercise period only in DL (−4 ± 5 and − 4 ± 5 mmHg). Afterward, diastolic BP increased in comparison to pre‐exercise at 60 and 90 min of the postexercise period (+2 ± 3 and + 3 ± 4 mmHg, *P*_interaction_ = 0.02), and mean BP increased at 90 min of the postexercise period (+3 ± 3 mmHg, *P*_interaction_ = 0.01) only in BL. HR increased significantly and similarly in all postexercise moments in comparison to pre‐exercise value (+4 ± 2 bpm, *P*_time_ <0.01). RPP increased significantly postexercise in both sessions (*P*_time_ <0.01). Regardless of the moment, RPP levels were higher in BL than DL (*P*_session_ = 0.05).

Three subjects did not complete the ABPM assessments in both experimental sessions. Thus, 17 subjects were included in the ABPM analyses presented in Table 4. No differences were observed for 24‐h, awake or asleep BPs, HRs, or RPPs between BL and DL sessions (all *p* > 0.05). However, RPP was significantly higher during the first 3 h of ABPM after BL than DL (11,117 ± 2055 vs. 10,189 ± 1046 mmHg × bpm, *p* = 0.05).

**TABLE 4:** Twenty‐four hour (24‐h), awake, asleep, and mean of the first 3 h of ambulatory blood pressures, heart rate and rate‐pressure‐product assessed after the bright light (BL) and dim light (DL) sessions.

	BL	DL	*p* Value
Systolic blood pressure (mmHg)			
24‐h	121 ± 9	121 ± 9	0.84
Awake	126 ± 10	126 ± 10	0.82
Asleep	111 ± 8	111 ± 9	0.54
Mean of the first 3 h	131 ± 9	131 ± 10	0.82
Diastolic blood pressure (mmHg)			
24‐h	70 ± 4	71 ± 5	0.88
Awake	75 ± 4	75 ± 5	0.63
Asleep	60 ± 6	61 ± 5	0.06
Mean of the first 3 h	81 ± 6	79 ± 6	0.28
Mean blood pressure (mmHg)			
24‐h	87 ± 5	87 ± 5	0.58
Awake	91 ± 5	91 ± 5	0.44
Asleep	77 ± 5	77 ± 5	0.42
Mean of the first 3 h	97 ± 5	96 ± 6	0.17
Heart rate (bpm)			
24‐h	71 ± 8	71 ± 7	0.46
Awake	75 ± 6	75 ± 6	0.42
Asleep	61 ± 9	61 ± 8	0.55
Mean of the first 3 h	86 ± 15	79 ± 8	0.07
Rate‐pressure‐product (mmHg × bpm)			
24‐h	8541 ± 821	8526 ± 858	0.52
Awake	9462 ± 942	9434 ± 823	0.65
Asleep	6735 ± 868	6592 ± 727	0.47
Mean of the first 3 h	11,117 ± 2055	10,189 ± 1046	**0.05**

*Note*: Data are shown as mean ± SD.

## 4 DISCUSSION

The purpose of this investigation was to evaluate whether BL (5000 lux) attenuates PEH magnitude and duration in young healthy men in comparison to DL (≤8 lux). The main findings were that BL exposure: (1) increased systolic BP levels before and after the exercise but did not affect its reduction at 30 min postexercise; (2) abolished the decrease in diastolic and mean BPs observed at 30 min after the exercise in the DL, and promoted an increase in these BPs at 60 and 90 min postexercise; (3) increased RPP levels before and after the exercise in the laboratory setting and for 3 h of ABPM measurements.

Although not the main objective of the present study, we observed that at resting conditions (baseline vs. pre‐exercise), BL did not induce any specific change in the cardiovascular parameters since the increase in diastolic and mean BPs as well as the decrease in HR and RPP were similar in the BL and DL sessions. The absence of change in resting BP and HR with BL exposure differs from expected based on the previous studies that reported an increase in these variables with BL (Sakakibara et al., 2000; Scheer et al., 2004; Yokoi et al., 2006). However, factors such as the duration of the exposition to the light and time of the day in which the experiments were conducted may explain this discrepancy. Studies that exposed the subjects to 3 h of BL reported an increase in BP compared to DL (Burgess et al., 2001; Yokoi et al., 2006), but no changes in BP were observed with light exposures of 30 (Saito et al., 1996) or 5 min (Shea et al., 1987). Considering the time of day, despite not directly investigating BP, a previous study found that HR and sympathetic modulation increased in response to light exposure (800 lux) in the early morning and nighttime, but this increase was blunted in the afternoon (Scheer et al., 1999). These aspects suggest that the absence of changes in cardiovascular parameters from baseline to pre‐exercise in the present study may be related to the short period (45 min) or the fact that the study was conducted in the early afternoon.

Considering the postexercise responses observed in the present investigation, the decrease of −3 ± 4 mmHg in systolic BP is consistent with the literature since a meta‐analysis about this topic found a reduction of 5 mmHg in men exercising on a cycle‐ergometer (Carpio‐Rivera et al., 2016). Additionally, HR increased postexercise and remained higher than pre‐exercise values for an extended period in the laboratory, as observed in previous studies (Brito et al., 2015; Forjaz et al., 1998). Thus, the aerobic exercise protocol used in the current study promoted the expected postexercise responses.

Regarding the effect of light on PEH, the results show that BL abolished PEH since the decrease in mean and diastolic BP 30 min after the exercise was not seen in the BL session, and these BPs were increased at 60 and 90 min after the exercise in this session. To the best of our knowledge, this is the first study to investigate the effect of BL on PEH, precluding any comparison with the literature. Although the underlying mechanisms of BL affecting BP and HR are out of the scope, the discussion can encourage others in the exercise physiology field. It has been shown in animals that BL stimulates retinal neurons and, via the retinohypothalamic tract, excites a particular set of suprachiasmatic nuclei neurons through a glutamatergic receptor‐mediated mechanism (Amir, 1992; de Vries et al., 1993; Meijer et al., 1996). Direct projections from the paraventricular nucleus (Buijs et al., 1999) and the nucleus of the solitary tract (Buijs et al., 2014) to the suprachiasmatic nucleus provide the neuroanatomical basis of its involvement in autonomic cardiovascular control. Indeed, a light‐mediated activation of the suprachiasmatic nuclei affected the neuronal output to the adrenal cortex utilizing the autonomic nervous system (Buijs et al., 1999), and light‐induced changes in HR were dependent on intact suprachiasmatic nuclei in rats (Scheer et al., 2001). Along this line, human studies have shown that BL exposure increases muscle sympathetic activity, although it did not change BP (Saito et al., 1996). Thus, it is possible to suppose that under BL, the decrease in muscle sympathetic nerve activity was blunted, in contrast to what has been observed after a session of exercise as a possible mechanism of PEH (Forjaz et al., 1999; Halliwill et al., 1996). This may have prevented the decrease in BP during the recovery period. This mechanism needs to be investigated in the future.

The absence of PEH when exercise is conducted under BL can have important clinical implications. First, PEH is important due to its hypotensive effect lasting many hours after an exercise session in subjects with high BP (Kenney & Seals, 1993). Thus, if BL also abolishes PEH in hypertensive populations, the acute benefit of exercise in this population can be lost with BL. A second possible implication of this finding is related to the effect of chronic training. Some researchers suggest that the adaptations to exercise training result from the sum of the acute responses to each exercise session (Brito, Fecchio, et al., 2018; da Nobrega, 2005). Along this line, if exposure to BL during the training sessions abolishes the BP decrease after the exercise, it may also preclude the chronic hypotensive effect of aerobic training. Since this is the first study on this subject (light effect on PEH), these findings must be further investigated. However, if the results were replicated in other populations and conditions, they may imply the recommendation for exercising under low‐intensity light when a BP decrease is desired.

Another thought‐provoking result of the present study was the greater RPP observed in BL during pre‐ and postexercise period and for the first 3 h of ambulatory monitoring. RPP is well‐known for being strongly associated with myocardial oxygen consumption and expressing cardiac work (Gobel et al., 1978). Additionally, RPP predicts adverse cardiovascular events (Jiang et al., 2023; White, 1999). Thus, RPP elevation for many hours after exercise may indicate an increased risk caused by BL exposure to adverse cardiovascular events in more vulnerable people, and avoiding BL during exercise sessions for these people would be recommended.

The present study has some strengths and limitations. As limitations: (1) the results are limited to subjects with similar characteristics to those included in this study and should not be directly extrapolated to women, elderly, and subjects with clinical conditions (i.e., hypertension); being necessary to replicate this study in other populations; and (2) the results are also limited to the characteristics of the experimental protocol as they could be different if the experiments were conducted at different times of day, if light is set to other intensities; and if exercise has different modality, intensity, duration, etc. The main strengths are: (1) all subjects exercised at the same workload and intensity, implying that the difference between BL and DL cannot be attributed to differences in the exercise; and (2) a standardized meal was provided before the experimental sessions, avoiding any interference of the hypotensive postprandial effect on the findings (Alfie, 2015).

## 5 CONCLUSION

In healthy adult men, BL exposure before, during, and after exercise abolished the postexercise decrease in diastolic and mean BPs and increased cardiac work compared to DL. Future studies need to reproduce these findings in people with hypertension to understand better the clinical relevance of BL management in exercise programs intended to promote cardiovascular health.

## AUTHOR CONTRIBUTIONS

GFO, TCM, and LCB conceived and designed research; GFO, TCM, JLB, and LA performed experiments; GFO analyzed data; GFO, LCB, SST, JCN, and CLMF interpreted experiments results; JCN, LCB, and CLMF obtained funding; GFO prepared figures; GFO and LCB drafted the manuscript; All authors edited, revised, and approved the final version of the manuscript.

## FUNDING INFORMATION

Fundação de Amparo à Pesquisa do Estado de São Paulo (FAPESP 2019/24327–5) to JCN; Fundação de Amparo à Pesquisa do Estado de São Paulo (FAPESP 2022/12605–3), Conselho Nacional de Desenvolvimento Científico e Tecnológico (CNPq 304436/2018–6; 302309/2022–5), and Coordenadoria de Aprimoramento Pessoal de Nível Superior (CAPES 0001) to CLMF; and Fundação de Amparo à Pesquisa do Estado de São Paulo (FAPESP 2018/05226–0), American Heart Association (grant 24CDA1267757), and OHSU Fellowship for Diversity in Research (OFDIR) to LCB.

## CONFLICT OF INTEREST STATEMENT

The authors report no conflict of interest.

## ETHICS STATEMENT

All subjects signed an informed written consent before enrollment. The study was approved by the Research Ethical Committee of the School of Physical Education and Sport of the University of São Paulo (3.742.479).
